# S‐adenosylmethionine: A metabolite critical to the regulation of autophagy

**DOI:** 10.1111/cpr.12891

**Published:** 2020-10-08

**Authors:** Yang Ouyang, Qi Wu, Juanjuan Li, Si Sun, Shengrong Sun

**Affiliations:** ^1^ Department of Breast and Thyroid Surgery Renmin Hospital of Wuhan University Wuhan China; ^2^ Department of Clinical Laboratory Renmin Hospital of Wuhan University Wuhan China

## Abstract

Autophagy is a mechanism that enables cells to maintain cellular homeostasis by removing damaged materials and mobilizing energy reserves in conditions of starvation. Although nutrient availability strongly impacts the process of autophagy, the specific metabolites that regulate autophagic responses have not yet been determined. Recent results indicate that S‐adenosylmethionine (SAM) represents a critical inhibitor of methionine starvation–induced autophagy. SAM is primarily involved in four key metabolic pathways: transmethylation, transsulphuration, polyamine synthesis and 5′‐deoxyadenosyl 5′‐radical–mediated biochemical transformations. SAM is the sole methyl group donor involved in the methylation of DNA, RNA and histones, modulating the autophagic process by mediating epigenetic effects. Moreover, the metabolites of SAM, such as homocysteine, glutathione, decarboxylated SAM and spermidine, also exert important influences on the regulation of autophagy. From our perspective, nuclear‐cytosolic SAM is a conserved metabolic inhibitor that connects cellular metabolic status and the regulation of autophagy. In the future, SAM might be a new target of autophagy regulators and be widely used in the treatment of various diseases.

## Graphical abstract

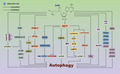

## 1 INTRODUCTION

Autophagy involves the lysosomal degradation of intracellular macromolecular components. It was first studied as a cellular response to starvation. However, autophagy is now a key regulator of cell metabolism, growth control, the balance between cell survival and cell death, and ageing.^1^ It is not surprising that autophagy plays a significant role in cellular homeostasis, thus affecting human health and disease.^2^ Autophagy is also involved in cell death and tumour suppression,^3^ neurodegeneration,^4^ ageing,^5^ inflammation,^6^ immunity^7^ and genome stability.^8^ In addition to starvation, autophagy is induced by many other disturbances, including hypoxia, metabolic disturbances, infiltration and oxidative stresses.^9^, ^10^, ^11^, ^12^ Autophagy is regulated by nutrient‐ and energy‐sensing pathways, including the well‐known energy regulators mammalian target of rapamycin (mTOR), AMPK (adenosine 5′‐monophosphate [AMP]–activated protein kinase) and AKT/protein kinase B.^13^ However, recent studies have shown that in addition to phosphorylation, other post‐translational modifications, including acetylation^14^ and methylation,^15^ of proteins may also impact autophagy by targeting these pathways, at least in part. These modifications are different from kinase‐mediated modulation. The methylation status of multiple regulatory factors and core components of the autophagy mechanism undergo changes, and the methylation of promoter CpG islands on DNA molecules or histone lysine residues is related to different aspects of autophagy.^16^

S‐adenosylmethionine (SAM) is a significant biological sulphonium compound that participates in a variety of biochemical processes. SAM is biosynthesized through the reaction of methionine with ATP that is catalysed by SAM synthetase or methionine adenosyltransferase (MAT). The reaction mainly occurs in cytosolic pools via the one‐carbon metabolism pathway that encompasses both the folate and methionine cycles.^17^ Several amino acids, namely serine, glycine and threonine, can initiate the folate cycle to facilitate the generation of SAM in the methionine cycle. SAM is considered to be the main methyl donor reagent for significant methylation reactions that occur in all living organisms, which are essential in cell differentiation and survival by regulating key metabolic pathways, including methylation and polyamine synthesis. Recent studies have shown that transcriptional regulation and epigenetic regulation are critical for the autophagic process.^18^ Mutations in core enzymes associated with one‐carbon metabolism and SAM are observed in cancer and are accompanied by aberrant methylation states.^19^ SAM is an important metabolite and can act as a nutrition, energy and stress sensor in vivo and in vitro, thus regulating autophagy. Moreover, SAM is involved in transmethylation, transsulphuration and polyamine synthesis and participates in the regulation of autophagy through different mechanisms. Thus, given that the autophagy response is influenced by the metabolic state of a cell, we concluded that the SAM level is a key regulator of autophagy and that regulating the SAM level can be a new therapeutic strategy for disease treatment.

## 2 SAM METABOLISM

### 2.1 Biosynthesis of SAM

**Figure 1:**
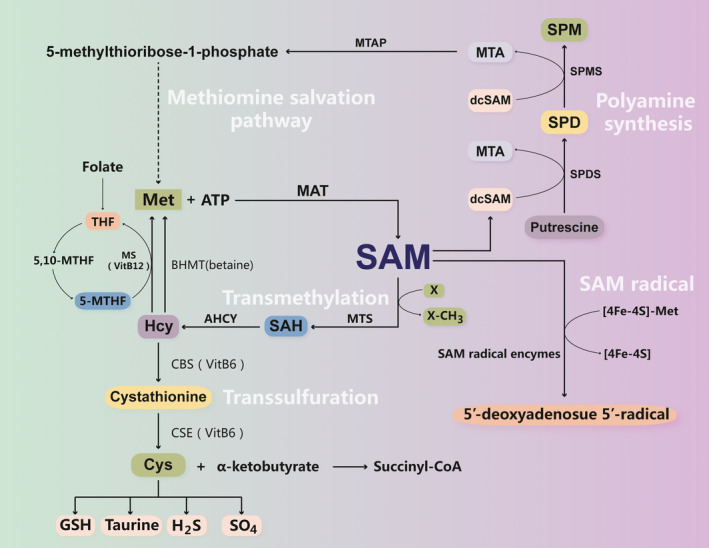
S‐adenosylmethionine (SAM) biosynthesis and metabolism. Methionine (Met) is converted into SAM by MAT in an ATP‐dependent process. SAM is linked to four key metabolic pathways: transmethylation, transsulphuration, polyamine synthesis and 5′‐deoxyadenosyl 5′‐radical–mediated biochemical transformations. In transmethylation, SAM donates its methyl group to many substrates, including DNA, RNA and proteins, which are catalysed by specific methyltransferases (MTs). After transmethylation reactions, SAM is converted to S‐adenosylhomocysteine (SAH), which is hydrolysed by a reversible enzyme called SAH hydrolase (AHCY) to form homocysteine (Hcy) and adenosine. Hcy has two fates: to be remethylated to regenerate Met or to enter the transsulphuration pathway. In transsulphuration, Hcy is first converted to cystathionine and then to cysteine (Cys) and α‐ketobutyrate, catalysed by the enzyme cystathionine β‐synthase (CBS). Then, Cys is converted to various sulphur‐containing molecules, including glutathione (GSH), taurine, hydrogen sulphide (H_2_S) and sulphate (SO_4_), which is catalysed by the enzyme cystathionase (CSE). Both CBS and CSE require vitamin B6 as a cofactor. α‐Ketobutyrate is converted to succinyl‐CoA, which is metabolized in the mitochondria. Hcy is remethylated to regenerate Met via two routes: the MS pathway and the betaine homocysteine S‐methyltransferase (BHMT) pathway. In the MS pathway, 5‐methyltetrahydrofolate (MTHF) donates a methyl group to Hcy, which requires folate and vitamin B12. In the BHMT route, Hcy uses betaine as a methyl donor. In polyamine synthesis, SAM is first decarboxylated to form decarboxylated SAM (dcSAM). Then, putrescine uses dcSAM as a propylamine donor, which is transformed to spermidine (SPD) and spermine (SPM), yielding 5′‐methylthioadenosine (MTA) as a by‐product. MTA is used to regenerate methionine through the methionine salvation pathway. In 5′‐deoxyadenosyl 5′‐radical–mediated biochemical transformations, SAM initiates various radical chemical reactions, which are catalysed by a large family of SAM radical enzymes. These enzymes share a CX_3_CX_2_C motif forming a characteristic [4Fe‐4S] cluster. SAM is converted to [4Fe‐4S]‐methionine and a 5′‐deoxyadenosyl 5′‐radical through binding to the [4Fe‐4S] cluster

S‐adenosylmethionine can be synthesized in the cytoplasm of every cell, especially hepatocytes.^20^ In liver, SAM synthesis is mainly derived from methionine, which accounts for half of the daily intake of amino acids.^21^, ^22^ Methionine is converted into SAM by MAT in an ATP‐dependent process (Figure 1).^21^ During this process, methionine is transformed to carry a sulphonium ion as a high‐energy reagent by combining with the adenosyl moiety of ATP; then, the activator transfers its methyl group to various substrates, including proteins, DNA, RNA and lipids.^23^ Methylation is an important protein post‐translational modification that not only regulates target gene transcription and expression but also controls the activity of various signalling pathways, including autophagy initiation pathways.^15^, ^24^ S‐adenosylhomocysteine (SAH) is the by‐product of the methyltransferase reaction and is transformed into homocysteine. Then, homocysteine is a precursor involved in the synthesis of cysteine and glutathione by the transsulphuration pathway. 5,10‐Methylenetetrahydrofolate (5,10‐methylene‐THF), which is derived from the folate cycle, can be irreversibly converted into 5‐methyl‐THF (5‐MTHF) by methylenetetrahydrofolate reductase (MTHFR), which donates its methyl group to homocysteine in the methionine synthase (MS) reaction to produce methionine and THF, requiring vitamin B12 as a cofactor.^25^ Sustaining the SAM cycle is dependent on intermediary metabolism and can be negatively affected by a diet lacking several essential nutrients, for example methionine, folate, vitamin B12 and vitamin B6. Impaired dietary supply, absorption, distribution, metabolism or enzymatic processing of these nutrients negatively impacts the cellular level of SAM.^26^, ^27^, ^28^, ^29^ Methionine restriction, for instance, results in a decrease in SAM level and extends the lifespan of different species by modulating autophagy.^30^

### 2.2 Degradation of SAM

S‐adenosylmethionine acts as a link between four key metabolic pathways, transmethylation, transsulphuration, polyamine synthesis and 5′‐deoxyadenosine 5′‐radical–mediated biochemical transformation (Figure 1), influencing the regulation of autophagy. In the following sections, the role of SAM in each of these four metabolic pathways will be described.

#### 2.2.1 Transmethylation

The most prominent metabolic function of SAM is its role as a methyl group donor in a variety of reactions catalysed by enzymes from the methyltransferase family.^31^ Indeed, more than 90% of the formed SAM molecules are consumed to sustain methylation reactions. In humans, most SAM is generated in liver, where more than half of the methionine from daily intake is metabolized and more than 85% of all methylation reactions occur. In transmethylation, SAM donates its methyl group to a large variety of receptor molecules. The reactions are catalysed by methyltransferases. In the human genome, more than 200 SAM‐dependent methyltransferases have been identified.^32^ Moreover, accumulating evidence has shown that several methyltransferases and demethylases are involved in regulating autophagy, such as H3K27 trimethylation by enhancer of zeste homolog 2 (EZH2).^33^ H3K9 dimethylation by G9a^34^ and non‐histone protein methylation are also associated with autophagy regulation.^15^ After delivery of the methyl group to the recipient compound, SAM is converted to SAH. In addition, it was confirmed that elevated levels of SAH and decreased levels of SAM inhibit transmethylation.^20^, ^21^ The ratio of SAM to SAH is generally considered to be a metabolic indicator that controls methylation in vivo, and a decrease in this ratio indicates a reduction in methylation ability. SAH is further converted to homocysteine (HCY), which is catalysed by SAH hydrolase (AHCY).^21^ It has been proven that HCY modulates autophagy in different cell types. However, the regulatory effect of HCY on autophagy has opposite effects (upregulation and downregulation) in different cell types and biological contexts.^35^ Then, HCY enters two metabolic pathways, remethylation to regenerate methionine or the transsulphuration pathway to generate cysteine and α‐ketobutyrate. Two enzymes are needed for the remethylation of HCY to form methionine, MS, which requires folate and vitamin B12, and betaine Hcy methyltransferase (BHMT), which requires betaine as a cofactor, which is a metabolite of choline.

#### 2.2.2 Transsulphuration

The transsulphuration pathway is another crucial catabolism process that links SAM to cysteine biosynthesis. Cysteine is generated from HCY via the transsulphuration reaction, and this process principally occurs in liver and lens.^36^ First, HCY is transformed into cystathionine by condensation with homoserine, and it is then converted to cysteine and α‐ketobutyrate. The above reactions are catalysed by cystathionine β‐synthase (CBS) and cystathionase (CSE), respectively, and both require vitamin B6 as a cofactor. Cysteine is converted to various sulphur‐containing molecules, including glutathione (GSH), taurine, sulphate (SO_4_) and hydrogen sulphide (H_2_S), whereas α‐ketobutyrate is converted to succinyl‐CoA, and this reaction mainly occurs in the mitochondria. SAM is a significant precursor of GSH, and the transsulphuration pathway is especially active in liver.^37^ GSH protects against oxidative damage in many tissues, and GSH depletion may induce autophagy.^38^ GSH redox homeostasis may be central to the proteostasis maintenance realized through autophagic regulation.^39^ In liver, SAM inhibits the activity of MTHFR and activates CBS.^40^, ^41^ Therefore, an increase in SAM reduces 5‐MTHF, the substrate for MS. Thus, the SAM level is the main control of the flux of sulphur‐containing molecules; when SAM is decreased, Hcy is directed to regenerate SAM through the remethylation pathway, and when the SAM level is increased, Hcy is directed to the transsulphuration pathway.

#### 2.2.3 Polyamine synthesis

S‐adenosylmethionine also participates in the synthesis of polyamines. Spermidine (SPD) and spermine (SPM) are the primary polyamines in mammalian cells. SPD and SPM have crucial effects on senescence, immunity and cancer.^42^, ^43^, ^44^ There is accumulating evidence showing that polyamines, including putrescine, SPD and SPM, are novel autophagy and longevity inducers.^42^, ^45^ In this pathway, SAM is decarboxylated and generates decarboxylated SAM (dcSAM), which is catalysed by the enzyme SAM decarboxylase; subsequently, the first aminopropyl group from dcSAM is added to putrescine to generate SPD, which is catalysed by SPD synthase, SPD is converted to SPM via the addition of the second aminopropyl group, which is catalysed by SPM synthase, and both the reactions yield 5′‐methylthioadenosine (MTA) as a by‐product.^46^, ^47^ Then, MTA is used to regenerate methionine through a methionine salvation pathway.^48^ In this pathway, MTA is first phosphorylated by MTA phosphorylase (MTAP), and then MTA is converted to adenine and 5‐methylthioribose‐1‐phosphate, which is further metabolized to methionine. When MTAP is deficient, endogenous MTA is unable to rescue methionine or adenine. This deficiency results in impaired polyamine biosynthesis and accumulation of dcSAM and MTA, and both inhibit the methylation reactions.^49^, ^50^, ^51^

#### 2.2.4 5’‐Deoxyadenosyl 5’‐radical–mediated biochemical transformations

Under anaerobic conditions, SAM also induces novel radical chemical reactions.^52^ This SAM radical enzyme family shares a CX3CX2C motif to form a characteristic [4Fe‐4S] cluster. SAM binds to the Fe in the [4Fe‐4S] cluster to produce [4Fe‐4S]‐methionine and a 5′‐deoxyadenosyl 5′‐radical. The 5′‐deoxyadenosyl 5′‐radical then removes a hydrogen atom from proteins, DNA or RNA to initiate the radical mechanism.^31^ There are some SAM radical enzymes recognized in humans: MOCS1, which is involved in molybdenum cofactor biosynthesis; LIAS, which is involved in lipoic acid biosynthesis; CDK5RAP1, which is involved in 2‐methylthio‐N(6)‐isopentenyladenosine biosynthesis; CDKAL1, which is involved in methylthio‐N(6)‐threonylcarbamoyl‐adenosine biosynthesis; TYW1, which is involved in wybutosine biosynthesis; ELP3, which is associated with 5‐methoxycarbonylmethyl uridine; and RSAD1 and viperin.^52^ Viperin (also known as radical SAM domain‐containing 2) is induced in various cells, including fibroblasts, hepatocytes and immune cells such as T cells and dendritic cells, playing an antiviral role and regulating cell signalling pathways or cellular metabolism.^53^, ^54^, ^55^, ^56^, ^57^

## 3 SAM AND AUTOPHAGY

**Figure 2:**
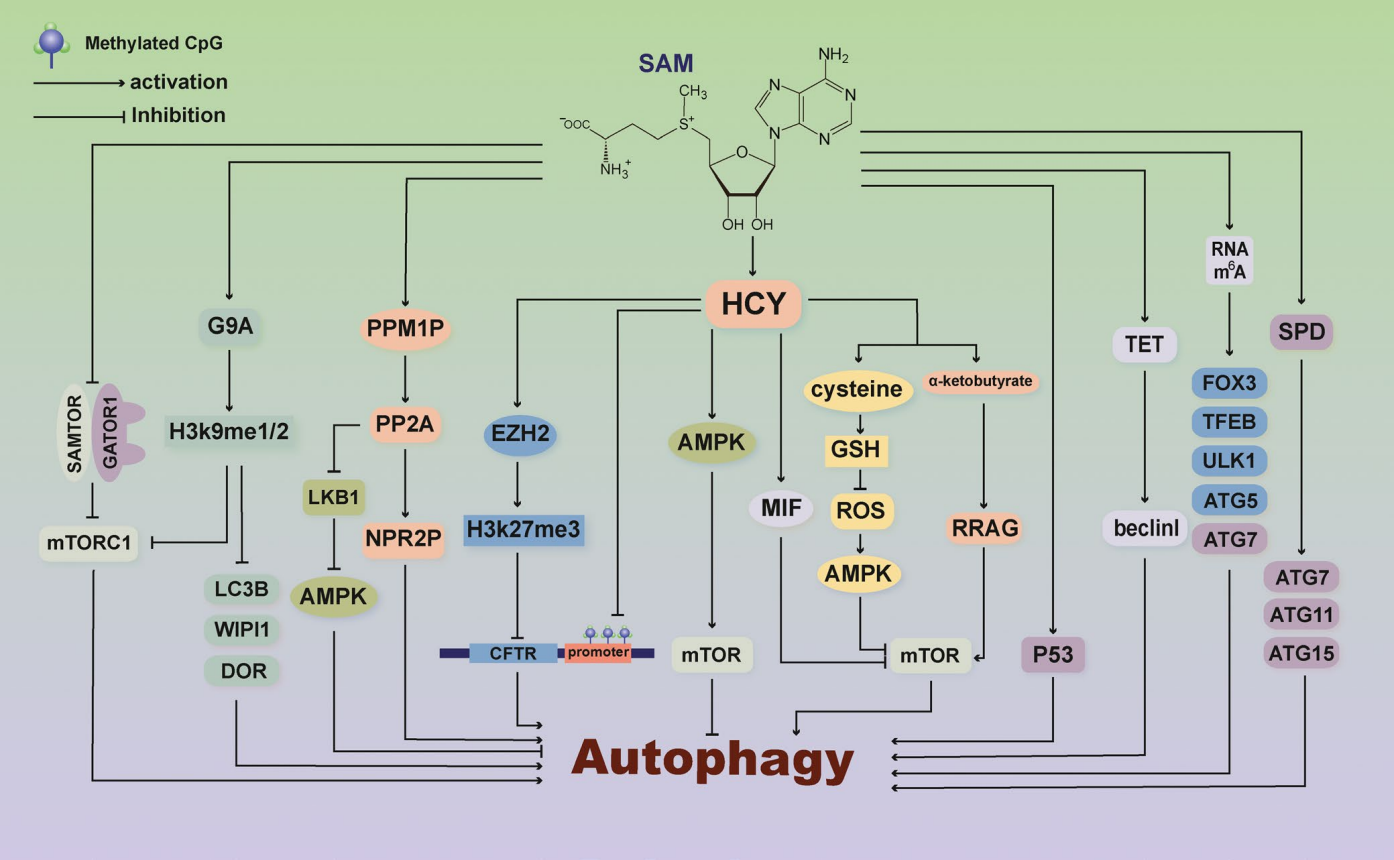
Working model for SAM‐mediated modulation of autophagy. SAM is an essential metabolite that acts as a high‐energy methyl donor for most methylation modifications of non‐histone, histone, DNA and RNA, which epigenetically affect autophagic flux. Moreover, SAM is the biosynthetic precursor for HCY and cysteine and other sulphur‐containing metabolites such as GSH. All of these roles significantly contribute to the modulation of autophagy. In addition, the SAM metabolite SPD has also been shown to be a physiological inducer of autophagy. Collectively, the metabolites of SAM and the key enzymes in SAM biosynthesis and metabolism influence the core autophagy machinery. S‐adenosylmethionine: SAM, homocysteine: HCY, glutathione: GSH, spermidine: SPD, AMP‐dependent protein kinase: AMPK, mechanistic target of rapamycin: mTOR, migration inhibitory factor: MIF, cystic fibrosis transmembrane conductance regulator: CFTR, mTOR complex 1: mTORC1, protein phosphatase 2A: PP2A, nitrogen permease regulating protein: NPR2P, ten‐eleven translocation methylcytosine dioxygenases: TET, N^6^‐methyladenosine: RNA methylation, m^6^A RNA methylation

Autophagy involves the lysosomal degradation pathway, which removes damaged and potentially harmful substances and replenishes energy reserves under starvation conditions to maintain cellular homeostasis. Although the regulation of autophagy is strongly dependent on the availability of nutrients, there are few descriptions of the specific metabolites that regulate autophagy. Recently, SAMTOR was found to link methionine and one‐carbon metabolism to mTORC1 signalling, thereby acting as a SAM sensor.^58^ SAMTOR inhibits mTORC1 signalling by interacting with GATOR1, the GTPase‐activating protein of RagA/B. SAM disrupts the SAMTOR‐GATOR1 complex by binding directly to SAMTOR. The decrease in intracellular SAM levels caused by methionine starvation promotes the binding of SAMTOR‐GATOR1, thereby inhibiting mTORC1 signalling and further inducing the autophagy response. This study shows that SAM regulates autophagy in the absence of a methylation event. Furthermore, a plethora of papers has revealed that SAM acts as a conserved metabolic switch, linking the cellular metabolic state to the modulation of autophagy via regulating molecule methylation^59^, ^60^, ^61^ and sulphuration^62^, ^63^, ^64^ and polyamine synthesis^65^, ^66^ (Figure 2).

## 4 SAM, as a methyl donor, epigenetically regulates autophagy

Methylation is a burgeoning post‐transcriptional and epigenetic modification research topic within autophagy research. It has been documented that protein methylation, including histone methylation and non‐histone methylation, is one of the post‐transcriptional and epigenetic modifications that regulates autophagy.^15^ DNA methylation transfers the methyl group from SAM to the fifth position of cytosine (5‐methylcytosine, 5‐mC) on the DNA strands, catalysed by DNA methyltransferases.^67^ DNA hypermethylation by DNA methyltransferase 1 (DNMT1) has been implicated as one of the cellular autophagy inducers during tumorigenesis.^68^ N^6^‐methyladenosine (m^6^A) RNA methylation, the most abundant modification found in messenger RNA (mRNA) in eukaryotic cells, is also reported to play roles in autophagy regulation.^69^ SAM is an essential metabolite that acts as a high‐energy methyl donor for most methylation modifications of proteins, DNA and RNA, which epigenetically affect autophagic flux.

### 4.1 Non‐histone methylation

Previous studies have shown that SAM production inhibits non‐nitrogen starvation (NNS)–induced autophagy through the activation of the methyltransferase PPM1P, which modifies the catalytic subunit of protein phosphatase 2A (PP2A).^70^ Furthermore, methylated PP2A promotes the dephosphorylation of nitrogen permease regulating protein 2P (NPR2P), a component of a conserved complex that regulates NNS autophagy^71^ and in the negative regulation of TORC1 signalling,^72^, ^73^ which leads to growth‐promoting and autophagy‐inhibiting functions. Collectively, SAM can regulate autophagy and TORC1 signalling by modulating the methylation status of PP2A.^70^ Moreover, in hepatocytes, SAM may methylate and activate PP2A and then block hepatocyte growth factor (HGF)–induced activation of LKB1/AMPK signalling.^61^ AMPK is a conversant activator of the autophagic response. Therefore, SAM may inhibit AMPK‐activated autophagy via the activation of PP2A. Taken together, cellular methionine and SAM levels modulate the methylation status of PP2A and then regulate autophagy through TORC1 or AMPK signalling pathways.

### 4.2 Histone methylation

S‐adenosylmethionine can regulate the histone methylation state as a metabolic coenzyme, which is linked to autophagy control. G9A is a methyltransferase catalysing monomethylation and dimethylation of H3K9 (H3K9me1 and H3K9me2) in chromatin.^74^, ^75^ SAM is also necessary for the stable interaction of G9a with H3K9M histones.^59^ Recent studies have demonstrated that G9A inhibition or silencing represses serine‐glycine biosynthesis by reducing H3K9me1 levels, which inhibits mTORC1 kinase activity and induces cell death in association with autophagy.^76^, ^77^ Under nutrient‐deficient conditions, G9A dissociates from the promoters in autophagosome genes such as LC3B, WIPI1 and DOR, with a subsequent H3K9me2 reduction at these sites, consequently resulting in transcriptional activation and autophagy stimulation.^34^ The conversion of serine to glycine leads to the formation of 5,10‐MTHF, a major contributor to SAM biosynthesis. Serine supplementation, but not glycine supplementation, can rescue the cell death phenotype inducted by G9A inhibition or silencing.^76^ This finding indicates that G9A inhibition or silencing represses the conversion of serine to glycine, which decreases the synthesis of 5,10‐MTHF and SAM. Therefore, a low level of SAM is sufficient to stimulate autophagy via G9A‐mediated epigenetic regulation.

### 4.3 DNA methylation

Among epigenetic modifications, DNA methylation is perhaps the best studied and the most common.^78^ DNA methylation of cytosine and adenine residues is carried out by SAM‐dependent methyltransferases.^67^ Recently, a study has shown that methionine deprivation results in a rapid decrease in intracellular SAM, inducing H3K4me3 and DNA demethylation and triggering p53 signalling in human embryonic stem cells.^60^ p53 is a critical component of stress signalling and adaptation, which can regulate autophagy.^79^ Furthermore, ten‐eleven translocation (TET) methylcytosine dioxygenases are enzymes involved in active and passive demethylation and gene activation. A recent study has shown that a decrease in TET methylcytosine dioxygenase 2 (TET2) promotes methylation of the Beclin 1 promoter and induces endothelial cell autophagy during the pathogenesis of atherosclerosis.^80^ TET3 also likely participates in the regulation of autophagy. TET3 is indirectly blocked by Sirtuin‐1 (SIRT1) upon the accelerated dimethylation of H3K9 and subsequent DNA demethylation and replication suppression, which plays an important role in regulating autophagy.^81^ These observations indicate that the SAM level can regulate the autophagic response via modulation of the DNA methylation state.

### 4.4 RNA methylation

RNA m^6^A modification consists of the methyltransferase complex, demethylases and RNA‐binding proteins, which adds another critical layer of epigenetic regulation to various cellular processes.^82^ A methyltransferase 'writer' complex contains METTL3 (Methyltransferase Like 3), METTL14 (Methyltransferase Like 14), and WTAP (WT1‐associated protein), for which SAM is a substrate, binding at methyl residues of the N^6^ atom of adenosine mRNA bases.^83^ ALKBH5 (alkB homolog 5) and FTO (fat mass and obesity‐associated protein) (erasers) reverse m^6^A modifications acting as RNA demethylases.^84^ YT521‐B homology (YTH) domains, including YTHDF1, YTHDF2, YTHDF3, YTHDC1 and YTHDC2 (readers), have a conserved m^6^A‐binding domain and preferentially recognize m^6^A in a methylation‐dependent manner.^82^ Recently, the role of m^6^A modification in autophagy was reported. Lin *et al* ^85^ reported that METTL3 depletion–mediated m^6^A level suppression promotes sorafenib resistance in liver cancer by activating the FOXO3‐mediated autophagy signalling pathway. Song *et al* ^86^ also found that silencing METTL3 augments autophagic flux in ischaemic heart disease and that this action is dependent on TFEB, a master regulator of lysosomal biogenesis and autophagy genes. In addition, inhibiting the RNA demethylase ALKBH5 has the opposite effect. Furthermore, Jin *et al* ^87^ demonstrated that FTO reverses the m^6^A modification of ULK1 transcripts, thus inhibiting mRNA degradation and upregulating the abundance of autophagy‐related protein ULK1 (unc‐51 like kinase 1), enhancing autophagy in HeLa cells. Wang *et al* ^88^ also reported that FTO positively modulates autophagy activation in adipocytes by targeting ATG5 and ATG7 in an m^6^A‐dependent and YTHDF2‐mediated manner. Moreover, m^6^A modifications of RNA during the regulation of autophagy are also found in the leucocytes of chronic kidney disease patients, bone‐derived mesenchymal stem cells and Leydig cells.^69^, ^89^, ^90^ Taken together, these findings reveal the functional importance of the m^6^A methylation machinery in autophagy. SAM, as a methyl donor, may be a regulator of autophagy through its targeting of the RNA m^6^A modification.

## 5 SAM, as the mediator of sulphur‐containing molecules, modulates autophagy

S‐adenosylmethionine is more than just a methyl donor. It is the biosynthetic precursor for HCY and cysteine and other sulphur‐containing metabolites such as GSH. All of these roles significantly contribute to the modulation of autophagy.^35^, ^63^, ^91^ HCY is a key determinant of the SAM metabolism cycle. The imbalance in HCY metabolism can modulate autophagy, which is involved in the pathogenesis of diseases such as cardiovascular diseases and neurological and psychiatric disorders.^92^ Moreover, the availability of SAM is a limiting factor of the synthesis of the endogenous antioxidant GSH.^93^ GSH and cysteine are critical for attenuating much of the oxidative damage generated.^91^ Cellular oxidative stress is one of the key factors in autophagic response modulation.^63^ Collectively, SAM modulates autophagy as a mediator of sulphur‐containing molecule levels.

HCY, a sulphhydryl‐containing amino acid, is an intermediate metabolite in methionine metabolism and is derived from SAM. SAM supplementation decreased HCY levels in diet‐induced hyperhomocysteinaemia in mouse plasma and brain.^94^ Moreover, higher plasma homocysteine levels (>12 μmol/L) are found in depressed patients, resulting in a significant decrease in cerebrospinal fluid SAM levels.^95^, ^96^ HCY inhibits the expression of cystic fibrosis transmembrane conductance regulator (CFTR) by two mechanisms, resulting in autophagy induction of hepatic cells both in vivo and in vitro.^62^ HCY enhances the DNA methylation of the CFTR promoter and promotes EZH2 expression, which is critical for the H3K27me3 modification on the CFTR promoter. CFTR is a cAMP‐activated anion channel expressed in the apical membrane of epithelial cells that contributes to ion balance and fluid transport in a number of epithelial cell types.^97^, ^98^ Previous reports have shown that the knockdown of CFTR can inhibit autophagy in prostate cancer cells.^99^ In addition, recent studies have demonstrated that HCY can induce autophagy through the macrophage migration inhibitory factor (MIF)/mTOR signalling pathway, which induces apoptosis/death in human umbilical vein endothelial cells as an independent risk factor for atherosclerosis.^100^ In contrast, HCY was found to inhibit autophagy in vascular smooth muscle cells via the AMPK/mTOR signalling pathway and then increase endothelin type B receptor expression.^101^ Moreover, HCY also activates mTORC1 to inhibit autophagy and form abnormal proteins in human neurons and mice.^102^ Thus, as an important metabolic intermediate of the SAM cycle, HCY has a role in regulating autophagy that varies by cell type and biological environment.

Furthermore, HCY also transforms into cysteine and α‐ketobutyrate by the transsulphuration pathway. Cysteine can be converted to various sulphur‐containing molecules, such as GSH, an important cellular antioxidant and free radical scavenger. SAM treatment in patients with cirrhosis leads to recovery of liver GSH levels.^103^ The response of the liver to endotoxaemia preserves hepatic SAM storage by increasing MAT gene expression and decreasing the utilization of SAM, leading to the inhibition of GSH synthesis.^104^ As a result, SAM is a key mediator and precursor of GSH. Mounting evidence suggests that GSH may play a role in the control of autophagy.^63^, ^105^ GSH deficiency triggers AMPK‐independent induction of autophagy.^106^ GSH is an intracellular antioxidant that helps to alleviate oxidative stress in proliferating cells. Nutrient deficiencies result in a significant decrease in intracellular GSH levels in cancer cells. This phenomenon relies on ABCC1‐mediated GSH degradation, as well as glutamate‐cysteine ligase inhibition and to a lesser extent on the formation of GSH‐protein mixed disulphides. All of these factors synergistically promote the regulation of autophagy by transforming the intracellular redox conditions to a more oxidizing state.^63^ In addition, α‐ketobutyrate inhibits ATP synthase in a manner that is dependent on downstream target of rapamycin (TOR) to prolong life of *C elegans*, which results in a decrease in ATP content and an increase in autophagy.^64^ Consist of this, α‐ketobutyrate reduction activates TORC1 by a Gtr1/RRAG‐independent mechanism in yeast.^107^, ^108^ Interestingly, in contrast with those observations, α‐ketobutyrate enhances the GTP load of RRAG proteins, thereby activating MTORC1 and inhibiting autophagy in mammalian cells.^109^, ^110^ It is worth noting that the difference in α‐ketobutyrate in the modulation of autophagy and the exact molecular mechanism remain to be explored in future studies. In conclusion, SAM, as a mediator of sulphur‐containing molecules, regulates the activity of TOR, which is a key modulator of autophagy.

## 6 SAM, as the precursor of polyamine, induces autophagy

S‐adenosylmethionine is converted to dcSAM by the enzyme adenosylmethionine decarboxylase. dcSAM is transformed into the polyamines SPM and SPD by donating an aminopropyl group. SPD and SPM are essential polycations that are involved in a wide variety of cellular processes, such as the regulation of chromatin structure, gene transcription and translation, DNA stabilization, signal transduction, cell growth and proliferation.^111^ Since dcSAM is unavailable for methyl transfer reactions and inhibits methylation reactions, such as DNA methylation,^112^ the steady‐state level of dcSAM is kept very low.^113^ The content of dcSAM can be increased greatly in mammalian cells by reducing polyamine synthesis. Treatment with difluoromethylornithine (DFMO), an inhibitor of ornithine decarboxylase (ODC) that suppresses production of putrescine, results in a huge increase in dcSAM, leading to dcSAM levels that are 3‐fold to 4‐fold greater than SAM levels.^114^, ^115^ Polyamine SPD has recently been shown to be a physiological inducer of autophagy in eukaryotic cells. SPD inhibits histone acetyltransferase activity, resulting in the upregulation of several autophagy‐related genes (ATG), such as ATG7, ATG11 and ATG15.^65^ When added to culture medium, SPD also directly induces autophagy in a manner that is independent of transcription. The mechanism has not yet been well elucidated but may be a consequence of enhanced deacetylation of essential autophagy‐related proteins such as ATG5 and ATG7.^42^ Furthermore, consumption of intracellular polyamines by inhibition of the biosynthetic enzyme ODC with DFMO inhibits autophagy induction in response to starvation or rapamycin treatment, with a decrease in the levels of LC3 and ATG5.^66^ Thus, deficiency in the biosynthesis of polyamines and cellular accumulation of dcSAM inhibits autophagy, which may regulate cell proliferation.

## 7 THERAPEUTIC STRATEGIES

The connection between SAM and autophagy was described above. As an important metabolite in the body, SAM plays an essential role in cell growth, apoptosis, death and differentiation.^116^ SAM dysregulation is involved in the occurrence and development of various diseases, such as neuropsychiatric disorders,^117^ cardiovascular disease,^118^ cancers,^119^ liver disease^22^, ^120^ and many other diseases.^121^, ^122^, ^123^ In the United States, SAM became better known after 1999 as an over‐the‐counter dietary supplement under the Dietary Supplement Health and Education Act and it has been reported to be an effective treatment for depression,^124^ cognitive deficits,^117^ chronic pain,^125^ osteoarthritis^126^ and liver support.^120^ Regulating the levels of SAM in vivo could improve the prognosis and the effects of treatment.^117^, ^120^, ^127^ Given the relationship between SAM and autophagy, new therapeutic strategies for SAM will be introduced in the following sections to provide new ideas for the treatment of diseases.

### 7.1 SAM as a treatment for inflammation‐induced diseases

Many experimental models and clinical trials have demonstrated the therapeutic effects of SAM in acute liver injury and liver fibrosis.^40^, ^104^, ^128^, ^129^ The hepatoprotective effects of SAM may be attributed to reduced hepatocytes oxidative stress and inflammatory responses through inhibiting nuclear factor‐kappa B (NF‐κB) nuclear translocation and activating nuclear factor erythroid 2–related factor (Nrf2).^130^ Lipopolysaccharide (LPS), an inducer of endotoxaemia, inactivates hepatic MAT.^131^ SAM treatment has been demonstrated to prevent the LPS‐induced serum tumour necrosis factor‐α (TNF‐α) increase in rats.^131^ In addition, SAM also plays a role in preserving the GSH store,^103^ which moderates LPS‐induced hepatotoxicity, thus preventing liver injury.^104^ GSH modulates autophagy by transforming the intracellular redox conditions to a more oxidizing state when the intracellular GSH levels in cancer cells decrease.^63^ We proposed that SAM treatment promotes the production of GSH and blocks the core autophagy machinery, which may play a role in the regulation of oxidative stress in cancer cells. In addition, SAM could have anti‐inflammatory and anti‐fibrotic effects on allergic airway inflammation and remodelling in a murine model of chronic asthma, most likely by reducing oxidative stress and inhibiting activation of the TGF‐β1 signalling pathway.^132^ SAM and MTA are also effective in preventing inflammation‐induced colon cancer in mice by reducing the expression of NF‐κB and STAT3, both of which are essential for promoting the survival and growth of cells during colon tumorigenesis.^133^ Previous studies have also demonstrated that SAM inhibits inflammation through the suppression of cytokines, such as IL‐6, IL‐8, IL‐10, TNF‐α and IFN‐γ.^130^, ^133^, ^134^ A randomized controlled trial of patients with Alzheimer's disease also demonstrated that higher plasma SAM levels are connected with a decrease in presenilin, IL‐6 and TNF‐α.^135^ Furthermore, SAM administration has been used in patients with hepatitis‐related cirrhosis^136^ and osteoarthritis^137^ by reducing inflammation. In summary, SAM treatment might inhibit autophagy by restoring intracellular GSH levels to reduce oxidative stress and exert an anti‐inflammatory effect by suppressing the secretion of inflammatory cytokines. Because SAM is a metabolite in the body and appears to be safe for regular use, it may be an ideal candidate for chemoprevention of inflammation‐induced diseases.

### 7.2 Targeted SAM treatments combined with immunotherapy

Emerging evidence from cancer immunotherapy clinical trials has highlighted an important role for T cells in mediating the elimination of tumours. While the results of immunotherapies have been encouraging in the context of haematological cancers and more recently in melanoma, targeting other solid cancers has been largely unsuccessful. Several factors, including metabolic competition in the tumour microenvironment, could suppress T‐cell function following infusion.^138^ Autophagy is essential in sustaining cellular homeostasis and plays a dual role in both tumour cell survival and death. Deletion of Atg5, Atg7 or Atg3 impairs peripheral T‐cell homeostasis and T‐cell survival and function.^139^, ^140^ Moreover, CD8+ T cells lacking Atg5 or Atg7 acquire an effector phenotype but are unable to survive or form functional memory T cells.^141^, ^142^, ^143^ These studies indicate a highly dynamic role for autophagy in T cell–mediated adaptive immune responses. DeVorkin *et al* showed that suppression of autophagy shifts T cells to a glycolytic phenotype and causes a reduction in SAM, which results in a global loss of H3K27me3 and concomitant gains in H3K4me3. As a consequence, autophagy‐deficient T cells transcriptionally reprogramme immune response genes to an effector memory state and enhance CD8+ T cell–mediated rejection of tumours.^144^ Sahin *et al* ^145^ also found that SAM increased the DNA methylation of FOXP3 and then diminished the suppression capacity of regulatory T cells (Treg cells) by decreasing the FOXP3 mRNA and protein levels in a dose‐dependent manner. Tregs have immunosuppressive functions and reverse cytotoxic T cell–mediated anti‐tumour immunity.^145^ Moreover, SAM treatment also reduces IL‐10 in Treg cells.^145^ This outcome highlights the therapeutic potential of SAM for use in immunotherapy in the future. In addition, SAM, as an important intermediate of the methionine cycle, participates in the folic acid cycle and affects one‐carbon metabolism. Serine is the major carbon donor to the one‐carbon metabolism pathway, which could directly regulate adaptive immunity by controlling T‐cell proliferative capacity.^146^, ^147^ SAM is a principal methyl donor and a key immune metabolite that affects one‐carbon metabolism and the autophagic afflux, playing an essential role in shaping adaptive T‐cell immunity and T‐cell homeostasis. Thus, targeted SAM treatments combined with immunotherapy might be a novel therapeutic strategy. However, the immunomodulatory effects of SAM on T cells in vivo remain to be explored in the future.

### 7.3 Metformin enhances anti‐tumour effects by regulating SAM levels

Metformin is an oral anti‐hyperglycaemic agent for type 2 diabetes (T2D) that promotes health through the modulation of epigenomic metabolism. Metformin has recently been shown to promote global DNA methylation in non‐cancerous, cancer‐prone and metastatic cancer cells through positively modulating the activity of AHCY (the SAH hydrolase) in an AMPK‐dependent manner and promoting the accumulation of SAM.^148^, ^149^ These results indicate that metformin‐induced DNA methylation plays a role in delaying or reversing the hallmarks of ageing or age‐related diseases, such as cancer. However, in contrast to those observations in mammalian cells, metformin disrupts the folate cycle, leading to a reduction in SAM levels in microbes and slowing ageing in *C elegans*.^150^ Moreover, metformin is also an inhibitor of the genes that participate in methionine biosynthesis transcription. It is interesting to note that limiting methionine in the diet can extend the lifespan of fruit flies and rodents. Bacteria in the human gut have a significant effect on nutrition and host biology. Metformin could also control the response of tumour therapy by modulating the tumour microenvironment,^151^ particularly in immunotherapy.^152^ Metformin might have potential therapeutic efficacy through attenuating microbial methionine biosynthesis and decreasing SAM levels^150^, ^153^ or affecting the composition of the intestinal microflora.^154^ Furthermore, it has been reported that metformin treatment modulates the autophagy mediators becn1, atg7, and LC3 II/I, activates AMPK and represses both the mTORC1 and mTORC2 signalling pathways, thus inducing autophagy^155^, ^156^ and playing a role in T2D treatment and anti‐tumour therapies.^157^, ^158^, ^159^ Therefore, metformin might regulate the metabolism of the epigenome by changing the level of SAM, altering mammalian physiology through its effects on gut microbiota, and playing an anti‐tumour role by activating autophagy. Hence, combined metformin and chemotherapy or immunotherapy can enhance anti‐tumour effects.

### 7.4 DFMO as a tumour chemopreventive agent

Difluoromethylornithine (DFMO), an inhibitor of ODC, antagonizes polyamine synthesis, resulting in a huge increase in dcSAM, leading to dcSAM levels that are 3‐fold 4‐fold greater than SAM levels.^114^, ^115^ DFMO has long been used for the treatment of sleeping sickness, since polyamines are essential for the proliferation of these protozoa.^160^ However, polyamines are also essential for mammalian cell proliferation. Recently, DFMO has been shown to strongly inhibit the autophagic response by decreasing the cellular levels of polyamine and increasing dcSAM. Moreover, dcSAM inhibits methylation reactions, such as DNA methylation,^112^ which may regulate cell proliferation. DFMO treatment inhibits the promotion and proliferation/progression stages of cancer both in vitro and vivo.^161^ Considering that DFMO is already approved by the US. Food and Drug Administration, DFMO could be a candidate agent in cancer treatment to control host autophagy. In addition, DFMO depletes tumours of polyamines and inhibits the growth of MYC‐deregulated tumours in animals.^162^, ^163^ Pre‐emptive blockade of polyamine synthesis effectively prevents neuroblastoma formation in the transgenic TH‐MYCN model.^162^ DFMO as a cancer chemopreventive agent that has attracted much interest in recent years since ODC is transactivated by the c‐myc oncogene in malignant tumours.^164^ Identical findings in multiple cancer initiation models have led to a focus on DFMO as a chemopreventive agent and a treatment in combination with other therapies for use in treating diseases, including neuroblastoma, colorectal neoplasia, pancreatic cancer, and skin cancer, in a large number of clinical settings.^161^, ^165^, ^166^, ^167^, ^168^, ^169^ In addition, polyamine depletion shapes the tumour microenvironment to change it from a tumour‐permissive microenvironment by suppressing immune system effectors to a microenvironment that promotes anti‐tumour immunity.^170^ DFMO treatment led to the recruitment of activated (IFNγ^+^) CD4^+^ T cells, CD8^+^ T cells and NK cells and an increase in tumour‐killing M1 macrophages in the tumour microenvironment of an ovarian cancer mouse model.^171^ Collectively, DFMO inhibits ODC, by serving as the rate‐limiting enzyme of polyamine biosynthesis in the SAM cycle, playing a role in tumour chemoprevention and tumour microenvironment regulation. DFMO that targets the tumour microenvironment (and immune effectors) could cooperate with immunotherapy to improve prognosis.

## 8 CONCLUSION

In summary, SAM is an important intermediate of the methionine cycle and primarily participates in four key metabolic pathways: transmethylation, transsulphuration, polyamine synthesis and 5′‐deoxyadenosyl 5′‐radical–mediated biochemical transformations. SAM can be synthesized in all cells through methionine metabolism. The most important function of SAM is to be the primary methyl donor involved in epigenetic regulation. In addition, SAM is also a precursor of (a) GSH, the main endogenous antioxidant; (b) polyamines, which participate in the modulation of transcription, translation, cell growth and apoptosis; and (c) 5′‐deoxyadenosyl 5′‐radical, which initiates a radical reaction. The metabolites of SAM and the key enzymes in SAM biosynthesis and metabolism influence the core autophagy machinery. The levels of SAM modulate autophagy accompanied by targeting the AMPK/mTOR pathway and regulating methyltransferase activity. SAM metabolism has received increasing attention from autophagy researchers and has provided new insights into the importance of SAM levels for autophagy regulation, thereby opening new avenues for autophagic control. Moreover, SAM treatment has been indicated that to inhibit autophagy and suppress the secretion of inflammatory cytokines, playing a role in ameliorating inflammatory cancers. There is evidence that some therapeutic strategies work against tumour cells by modulating the levels of SAM, GSH or dcSAM and then stimulating autophagy. These therapeutic methods could also be combined with other treatments, such as chemotherapy and immunotherapy, and may provide a new effective therapeutic method to improve prognosis. However, new clinical trials are necessary to unambiguously establish the benefit of these methods in tumour therapy. Future research may investigate how the formation of the transcriptional complex and epigenetic alterations mediate the relationships among SAM accumulation, hypermethylation of DNA and histones, and autophagy repression. The therapeutic potential of SAM in tumours and other diseases should also be investigated.

## CONFLICT OF INTEREST

No potential conflicts of interest were disclosed.

## AUTHORS’ CONTRIBUTIONS

Q. Wu, Y. Ouyang and SR. Sun conceived and designed the study. S. Sun, Y. Ouyang and Q. Wu. contributed to acquisition of data. Y. Ouyang, Q. Wu and JJ Li analysed and interpreted the data. Y. Ouyang, Q. Wu and SR. Sun wrote, reviewed and revised the manuscript.

## Data Availability

Research data are not shared.

## References

[1] White E , Mehnert JM , Chan CS . Autophagy, metabolism, and cancer. Clin Cancer Res. 2015;21(22):5037‐5046. 26567363 10.1158/1078-0432.CCR-15-0490 PMC4646728

[2] Saha S , Panigrahi DP , Patil S , Bhutia SK . Autophagy in health and disease: a comprehensive review. Biomed Pharmacother. 2018;104:485‐495. 29800913 10.1016/j.biopha.2018.05.007

[3] Levy JMM , Towers CG , Thorburn A . Targeting autophagy in cancer. Nat Rev Cancer. 2017;17(9):528‐542. 28751651 10.1038/nrc.2017.53 PMC5975367

[4] He CC , Klionsky DJ . Autophagy and neurodegeneration. ACS Chem Biol. 2006;1(4):211‐213. 17163674 10.1021/cb600182h

[5] Abdellatif M , Sedej S , Carmona‐Gutierrez D , Madeo F , Kroemer G . Autophagy in cardiovascular aging. Circ Res. 2018;123(7):803‐824. 30355077 10.1161/CIRCRESAHA.118.312208

[6] Matsuzawa‐Ishimoto Y , Hwang S , Cadwell K . Autophagy and inflammation. Annu Rev Immunol. 2018;36(1):73‐101. 29144836 10.1146/annurev-immunol-042617-053253

[7] Jang YJ , Kim JH , Byun S . Modulation of autophagy for controlling immunity. Cells. 2019;8(2):138. 10.3390/cells8020138 PMC6406335 30744138

[8] Hewitt G , Korolchuk VI . Repair, reuse, recycle: the expanding role of autophagy in genome maintenance. Trends Cell Biol. 2017;27(5):340‐351. 28011061 10.1016/j.tcb.2016.11.011

[9] Tamura N , Kageyama S , Komatsu M , Waguri S . Hyperosmotic Stress Induces Unconventional Autophagy Independent of the Ulk1 Complex. Molecular and Cellular Biology. 2019;39 (16). 10.1128/mcb.00024-19 PMC6664608 31160490

[10] Cosin‐Roger J , Simmen S , Melhem H , et al. Hypoxia ameliorates intestinal inflammation through NLRP3/mTOR downregulation and autophagy activation. Nat Commun. 2017;8(1):98. 28740109 10.1038/s41467-017-00213-3 PMC5524634

[11] Yang Y , Zhao C , Yang P , Wang X , Wang L , Chen A . Autophagy in cardiac metabolic control: novel mechanisms for cardiovascular disorders. Cell Biol Int. 2016;40(9):944‐954. 27191043 10.1002/cbin.10626

[12] Vikram A , Anish R , Kumar A , Tripathi DN , Kaundal RK . Oxidative stress and autophagy in metabolism and longevity. Oxid Med Cell Longev. 2017;2017:3451528. 28337248 10.1155/2017/3451528 PMC5350394

[13] Rybstein MD , Bravo‐San Pedro JM , Kroemer G , Galluzzi L . The autophagic network and cancer. Nat Cell Biol. 2018;20(3):243‐251. 29476153 10.1038/s41556-018-0042-2

[14] Eisenberg T , Schroeder S , Andryushkova A , et al. Nucleocytosolic depletion of the energy metabolite acetyl‐coenzyme a stimulates autophagy and prolongs lifespan. Cell Metab. 2014;19(3):431‐444. 24606900 10.1016/j.cmet.2014.02.010 PMC3988959

[15] Li R , Wei X , Jiang D‐S . Protein methylation functions as the posttranslational modification switch to regulate autophagy. Cell Mol Life Sci. 2019;76(19):3711‐3722. 31222372 10.1007/s00018-019-03161-x PMC11105718

[16] Baek SH , Kim KI . Epigenetic control of autophagy: nuclear events gain more attention. Mol Cell. 2017;65(5):781‐785. 28257699 10.1016/j.molcel.2016.12.027

[17] Yang M , Vousden KH . Serine and one‐carbon metabolism in cancer. Nat Rev Cancer. 2016;16(10):650‐662. 27634448 10.1038/nrc.2016.81

[18] Shin H‐JR , Kim H , Kim KI , Baek SH . Epigenetic and transcriptional regulation of autophagy. Autophagy. 2016;12(11):2248‐2249. 27487449 10.1080/15548627.2016.1214780 PMC5103355

[19] Wu Q , Li J , Sun S , Chen X , Zhang H , Li B , Sun S . YAP/TAZ‐mediated activation of serine metabolism and methylation regulation is critical for LKB1‐deficient breast cancer progression. Bioscience Reports. 2017;37 (5). 10.1042/bsr20171072 PMC5653917 28931725

[20] Mato JM , Martinez‐Chantar ML , Lu SC . S‐adenosylmethionine metabolism and liver disease. Ann Hepatol. 2013;12(2):183‐189. 23396728 PMC4027041

[21] Lu SC . S‐Adenosylmethionine. Int J Biochem Cell Biol. 2000;32(4):391‐395. 10762064 10.1016/s1357-2725(99)00139-9

[22] Lu SC , Mato JM . S‐adenosylmethionine in liver health, injury, and cancer. Physiol Rev. 2012;92(4):1515‐1542. 23073625 10.1152/physrev.00047.2011 PMC3698976

[23] Struck AW , Thompson ML , Wong LS , Micklefield J . S‐adenosyl‐methionine‐dependent methyltransferases: highly versatile enzymes in biocatalysis, biosynthesis and other biotechnological applications. ChemBioChem. 2012;13(18):2642‐2655. 23180741 10.1002/cbic.201200556

[24] Hu L‐F . Epigenetic regulation of autophagy. Adv Exp Med Biol. 2019;1206:221‐236. 31776988 10.1007/978-981-15-0602-4_11

[25] Scott JM , Weir DG . The methyl folate trap. A physiological response in man to prevent methyl group deficiency in kwashiorkor (methionine deficiency) and an explanation for folic‐acid induced exacerbation of subacute combined degeneration in pernicious anaemia. Lancet. 1981;2(8242):337‐340. 6115113 10.1016/s0140-6736(81)90650-4

[26] Sanderson SM , Gao X , Dai Z , Locasale JW . Methionine metabolism in health and cancer: a nexus of diet and precision medicine. Nat Rev Cancer. 2019;19(11):625‐637. 31515518 10.1038/s41568-019-0187-8

[27] Mentch SJ , Mehrmohamadi M , Huang L , et al. Histone methylation dynamics and gene regulation occur through the sensing of one‐carbon metabolism. Cell Metab. 2015;22(5):861‐873. 26411344 10.1016/j.cmet.2015.08.024 PMC4635069

[28] Smith AD , Warren MJ , Refsum H . Vitamin B. Adv Food Nutr Res. 2018;83:215‐279. 29477223 10.1016/bs.afnr.2017.11.005

[29] Belardo A , Gevi F , Zolla L . The concomitant lower concentrations of vitamins B6, B9 and B12 may cause methylation deficiency in autistic children. J Nutr Biochem. 2019;70:38‐46. 31151052 10.1016/j.jnutbio.2019.04.004

[30] Parkhitko AA , Jouandin P , Mohr SE , Perrimon N . Methionine metabolism and methyltransferases in the regulation of aging and lifespan extension across species. Aging Cell. 2019;18(6):e13034. 31460700 10.1111/acel.13034 PMC6826121

[31] Fontecave M , Atta M , Mulliez E . S‐adenosylmethionine: nothing goes to waste. Trends Biochem Sci. 2004;29(5):243‐249. 15130560 10.1016/j.tibs.2004.03.007

[32] Petrossian TC , Clarke SG . Uncovering the human methyltransferasome. Mol Cell Proteomics. 2011;10(1):M110.000976. 10.1074/mcp.M110.000976 PMC3013446 20930037

[33] Wei F‐Z , Cao Z , Wang X , et al. Epigenetic regulation of autophagy by the methyltransferase EZH2 through an MTOR‐dependent pathway. Autophagy. 2015;11(12):2309‐2322. 26735435 10.1080/15548627.2015.1117734 PMC4835210

[34] Artal‐Martinez de Narvajas A , Gomez TS , Zhang JS , et al. Epigenetic regulation of autophagy by the methyltransferase G9a. Mol Cell Biol. 2013;33(20):3983‐3993. 23918802 10.1128/MCB.00813-13 PMC3811684

[35] Wang M , Liang X , Cheng M , et al. Homocysteine enhances neural stem cell autophagy in in vivo and in vitro model of ischemic stroke. Cell Death Dis. 2019;10(8):561. 31332165 10.1038/s41419-019-1798-4 PMC6646339

[36] Kredich NM . Biosynthesis of Cysteine. EcoSal Plus. 2008;3 (1). 10.1128/ecosalplus.3.6.1.11 26443742

[37] Lu SC . Regulation of glutathione synthesis. Mol Aspects Med. 2009;30(1–2):42‐59. 18601945 10.1016/j.mam.2008.05.005 PMC2704241

[38] Sun Y , Zheng Y , Wang C , Liu Y . Glutathione depletion induces ferroptosis, autophagy, and premature cell senescence in retinal pigment epithelial cells. Cell Death & Disease. 2018;9 (7). 10.1038/s41419-018-0794-4 PMC6037763 29988039

[39] Guerrero‐Gómez D , Mora‐Lorca JA , Sáenz‐Narciso B , et al. Loss of glutathione redox homeostasis impairs proteostasis by inhibiting autophagy‐dependent protein degradation. Cell Death Differ. 2019;26(9):1545‐1565. 30770874 10.1038/s41418-018-0270-9 PMC6748101

[40] Mato JM , Alvarez L , Ortiz P , Pajares MA . S‐adenosylmethionine synthesis: molecular mechanisms and clinical implications. Pharmacol Ther. 1997;73(3):265‐280. 9175157 10.1016/s0163-7258(96)00197-0

[41] Prudova A . Modulation of homocysteine metabolism and redox homeostasis via regulation of cystathionine beta‐synthase. The University of Nebraska‐Lincoln; 2006 https://digitalcommons.unl.edu/dissertations/AAI3208125

[42] Madeo F , Eisenberg T , Buttner S , Ruckenstuhl C , Kroemer G . Spermidine: a novel autophagy inducer and longevity elixir. Autophagy. 2010;6(1):160‐162. 20110777 10.4161/auto.6.1.10600

[43] Madeo F , Eisenberg T , Pietrocola F , Kroemer G . Spermidine in health and disease. Science. 2018;359:6374. 10.1126/science.aan2788 29371440

[44] Pietrocola F , Castoldi F , Kepp O , Carmona‐Gutierrez D , Madeo F , Kroemer G . Spermidine reduces cancer‐related mortality in humans. Autophagy. 2019;15(2):362‐365. 30354939 10.1080/15548627.2018.1539592 PMC6333461

[45] Zhou S , Gu J , Liu R , et al. Spermine alleviates acute liver injury by inhibiting liver‐resident macrophage pro‐inflammatory response through ATG5‐dependent autophagy. Front Immunol. 2018;9:948. 29770139 10.3389/fimmu.2018.00948 PMC5940752

[46] Soda K . Polyamine metabolism and gene methylation in conjunction with one‐carbon metabolism. Int J Mol Sci. 2018;19(10):3106. 10.3390/ijms19103106 PMC6213949 30309036

[47] Casero RA Jr , Murray Stewart T , Pegg AE . Polyamine metabolism and cancer: treatments, challenges and opportunities. Nat Rev Cancer. 2018; 18:681‐695. 30181570 10.1038/s41568-018-0050-3 PMC6487480

[48] Pegg AE , Williams‐Ashman HG . Phosphate‐stimulated breakdown of 5'‐methylthioadenosine by rat ventral prostate. Biochem J. 1969;115(2):241‐247. 5378381 10.1042/bj1150241 PMC1185095

[49] Heby O . DNA methylation and polyamines in embryonic development and cancer. Int J Dev Biol. 1995;39(5):737‐757. 8645558

[50] Kujubu DA , Stimmel JB , Law RE , Herschman HR , Clarke S . Early responses of PC‐12 cells to NGF and EGF: effect of K252a and 5'‐methylthioadenosine on gene expression and membrane protein methylation. J Neurosci Res. 1993;36(1):58‐65. 8230321 10.1002/jnr.490360107

[51] Dante R , Arnaud M , Niveleau A . Effects of 5'deoxy‐5'‐methylthioadenosine on the metabolism of S‐adenosyl methionine. Biochem Biophys Res Commun. 1983;114(1):214‐221. 6309166 10.1016/0006-291x(83)91615-7

[52] Landgraf BJ , McCarthy EL , Booker SJ . Radical S‐adenosylmethionine enzymes in human health and disease. Annu Rev Biochem. 2016;85:485‐514. 27145839 10.1146/annurev-biochem-060713-035504

[53] Dumbrepatil AB , Ghosh S , Zegalia KA , et al. Viperin interacts with the kinase IRAK1 and the E3 ubiquitin ligase TRAF6, coupling innate immune signaling to antiviral ribonucleotide synthesis. J Biol Chem. 2019;294(17):6888‐6898. 30872404 10.1074/jbc.RA119.007719 PMC6497957

[54] Steinbusch MMF , Caron MMJ , Surtel DAM , et al. The antiviral protein viperin regulates chondrogenic differentiation via CXCL10 protein secretion. J Biol Chem. 2019;294(13):5121‐5136. 30718282 10.1074/jbc.RA119.007356 PMC6442052

[55] Wang X , Hinson ER , Cresswell P . The interferon‐inducible protein viperin inhibits influenza virus release by perturbing lipid rafts. Cell Host Microbe. 2007;2(2):96‐105. 18005724 10.1016/j.chom.2007.06.009

[56] Saitoh T , Satoh T , Yamamoto N , et al. Antiviral protein viperin promotes toll‐like receptor 7‐ and toll‐like receptor 9‐mediated type I interferon production in plasmacytoid dendritic cells. Immunity. 2011;34(3):352‐363. 21435586 10.1016/j.immuni.2011.03.010

[57] Qiu LQ , Cresswell P , Chin KC . Viperin is required for optimal Th2 responses and T‐cell receptor‐mediated activation of NF‐kappa B and AP‐1. Blood. 2009;113(15):3520‐3529. 19047684 10.1182/blood-2008-07-171942

[58] Gu X , Orozco JM , Saxton RA , et al. SAMTOR is an S‐adenosylmethionine sensor for the mTORC1 pathway. Science. 2017;358(6364):813‐818. 29123071 10.1126/science.aao3265 PMC5747364

[59] Jayaram H , Hoelper D , Jain SU , et al. S‐adenosyl methionine is necessary for inhibition of the methyltransferase G9a by the lysine 9 to methionine mutation on histone H3. Proc Natl Acad Sci USA. 2016;113(22):6182‐6187. 27185940 10.1073/pnas.1605523113 PMC4896705

[60] Shiraki N , Shiraki Y , Tsuyama T , et al. Methionine metabolism regulates maintenance and differentiation of human pluripotent stem cells. Cell Metab. 2014;19(5):780‐794. 24746804 10.1016/j.cmet.2014.03.017

[61] Martinez‐Chantar ML , Vazquez‐Chantada M , Garnacho M , et al. S‐adenosylmethionine regulates cytoplasmic HuR via AMP‐activated kinase. Gastroenterology. 2006;131(1):223‐232. 16831604 10.1053/j.gastro.2006.04.019

[62] Yang A , Jiao Y , Yang S , et al. Homocysteine activates autophagy by inhibition of CFTR expression via interaction between DNA methylation and H3K27me3 in mouse liver. Cell Death Dis. 2018;9(2):169. 29415998 10.1038/s41419-017-0216-z PMC5833451

[63] Desideri E , Filomeni G , Ciriolo MR . Glutathione participates in the modulation of starvation‐induced autophagy in carcinoma cells. Autophagy. 2012;8(12):1769‐1781. 22964495 10.4161/auto.22037 PMC3541287

[64] Chin RM , Fu X , Pai MY , et al. The metabolite alpha‐ketoglutarate extends lifespan by inhibiting ATP synthase and TOR. Nature. 2014;510(7505):397‐401. 24828042 10.1038/nature13264 PMC4263271

[65] Eisenberg T , Knauer H , Schauer A , et al. Induction of autophagy by spermidine promotes longevity. Nat Cell Biol. 2009;11(11):1305‐1314. 19801973 10.1038/ncb1975

[66] Vanrell MC , Cueto JA , Barclay JJ , et al. Polyamine depletion inhibits the autophagic response modulating *Trypanosoma cruzi* infectivity. Autophagy. 2013;9(7):1080‐1093. 23697944 10.4161/auto.24709 PMC3722317

[67] Jones PA . Functions of DNA methylation: islands, start sites, gene bodies and beyond. Nat Rev Genet. 2012;13(7):484‐492. 22641018 10.1038/nrg3230

[68] Wong KK . DNMT1: A key drug target in triple‐negative breast cancer. Semin Cancer Biol. 2020. 10.1016/j.semcancer.2020.05.010 32461152

[69] Chen Y , Wang J , Xu D , et al. m6A mRNA methylation regulates testosterone synthesis through modulating autophagy in Leydig cells. Autophagy. 2020;1–19. 10.1080/15548627.2020.1720431 PMC8007139 31983283

[70] Sutter BM , Wu X , Laxman S , Tu BP . Methionine inhibits autophagy and promotes growth by inducing the SAM‐responsive methylation of PP2A. Cell. 2013;154(2):403‐415. 23870128 10.1016/j.cell.2013.06.041 PMC3774293

[71] Wu X , Tu BP , Subramani S . Selective regulation of autophagy by the Iml1‐Npr2‐Npr3 complex in the absence of nitrogen starvation. Mol Biol Cell. 2011;22(21):4124‐4133. 21900499 10.1091/mbc.E11-06-0525 PMC3204073

[72] Laxman S , Sutter BM , Lei Shi TuBP . Npr2 inhibits TORC1 to prevent inappropriate utilization of glutamine for biosynthesis of nitrogen‐containing metabolites. Sci Signal. 2014;7(356):ra120. 25515537 10.1126/scisignal.2005948 PMC4427238

[73] Liu Y , Okamoto K . The TORC1 signaling pathway regulates respiration‐induced mitophagy in yeast. Biochem Bioph Res Commun. 2018;502(1):76‐83. 10.1016/j.bbrc.2018.05.123 29787763

[74] Shankar SR , Bahirvani AG , Rao VK , Bharathy N , Ow JR , Taneja R . G9a, a multipotent regulator of gene expression. Epigenetics. 2013;8(1):16‐22. 23257913 10.4161/epi.23331 PMC3549875

[75] Poulard C , Bittencourt D , Wu DY , Hu Y , Gerke DS , Stallcup MR . A post‐translational modification switch controls coactivator function of histone methyltransferases G9a and GLP. EMBO Rep. 2017;18(8):1442‐1459. 28615290 10.15252/embr.201744060 PMC5538762

[76] Ding J , Li T , Wang X , et al. The histone H3 methyltransferase G9A epigenetically activates the serine‐glycine synthesis pathway to sustain cancer cell survival and proliferation. Cell Metab. 2013;18(6):896‐907. 24315373 10.1016/j.cmet.2013.11.004 PMC3878056

[77] Yin C , Ke X , Zhang R , et al. G9a promotes cell proliferation and suppresses autophagy in gastric cancer by directly activating mTOR. FASEB J. 2019;33(12):14036‐14050. 31647887 10.1096/fj.201900233RR

[78] Moore LD , Le T , Fan G . DNA methylation and its basic function. Neuropsychopharmacology. 2013;38(1):23‐38. 22781841 10.1038/npp.2012.112 PMC3521964

[79] White E . Autophagy and p53. Cold Spring Harb Perspect Med. 2016;6(4):a026120. 27037419 10.1101/cshperspect.a026120 PMC4817743

[80] Peng J , Yang Q , Li A‐F , et al. Tet methylcytosine dioxygenase 2 inhibits atherosclerosis via upregulation of autophagy in ApoE‐/‐ mice. Oncotarget. 2016;7(47):76423‐76436. 27821816 10.18632/oncotarget.13121 PMC5363520

[81] Zhao H , Yang L , Cui H . SIRT1 regulates autophagy and diploidization in parthenogenetic haploid embryonic stem cells. Biochem Biophys Res Commun. 2015;464(4):1163‐1170. 26208454 10.1016/j.bbrc.2015.07.098

[82] Lan Q , Liu PY , Haase J , Bell JL , Hüttelmaier S , Liu T . The critical role of RNA mA methylation in cancer. Can Res. 2019;79(7):1285‐1292. 10.1158/0008-5472.CAN-18-2965 30894375

[83] Wang X , Huang J , Zou T , Yin P . Human mA writers: Two subunits, 2 roles. RNA Biol. 2017;14(3):300‐304. 28121234 10.1080/15476286.2017.1282025 PMC5367249

[84] Meyer KD , Jaffrey SR . Rethinking mA readers, writers, and erasers. Annu Rev Cell Dev Biol. 2017;33:319‐342. 28759256 10.1146/annurev-cellbio-100616-060758 PMC5963928

[85] Lin Z , Niu Y , Wan A , et al. RNA m6A methylation regulates sorafenib resistance in liver cancer through FOXO3‐mediated autophagy. EMBO J. 2020;39(12):e103181. 32368828 10.15252/embj.2019103181 PMC7298296

[86] Song H , Feng X , Zhang H , et al. METTL3 and ALKBH5 oppositely regulate mA modification of mRNA, which dictates the fate of hypoxia/reoxygenation‐treated cardiomyocytes. Autophagy. 2019;15(8):1419‐1437. 30870073 10.1080/15548627.2019.1586246 PMC6613905

[87] Jin S , Zhang X , Miao Y , et al. m6A RNA modification controls autophagy through upregulating ULK1 protein abundance. Cell Res. 2018;28(9):955‐957. 30046135 10.1038/s41422-018-0069-8 PMC6123428

[88] Wang X , Wu R , Liu Y , et al. m(6)A mRNA methylation controls autophagy and adipogenesis by targeting Atg5 and Atg7. Autophagy. 2020;16:1221‐1235. 31451060 10.1080/15548627.2019.1659617 PMC7469583

[89] Li G , Song Y , Liao Z , et al. Bone‐derived mesenchymal stem cells alleviate compression‐induced apoptosis of nucleus pulposus cells by N6 methyladenosine of autophagy. Cell Death Dis. 2020;11(2):103. 32029706 10.1038/s41419-020-2284-8 PMC7005291

[90] Wang C‐Y , Lin T‐A , Ho M‐Y , et al. Regulation of autophagy in leukocytes through RNA N‐adenosine methylation in chronic kidney disease patients. Biochem Bioph Res Commun. 2020;527(4):953‐959. 10.1016/j.bbrc.2020.04.138 32439179

[91] Paul BD , Sbodio JI , Snyder SH . Cysteine metabolism in neuronal redox homeostasis. Trends Pharmacol Sci. 2018;39(5):513‐524. 29530337 10.1016/j.tips.2018.02.007 PMC5912966

[92] Skovierova H , Vidomanova E , Mahmood S , et al. The molecular and cellular effect of homocysteine metabolism imbalance on human health. Int J Mol Sci. 2016;17(10):1733. 10.3390/ijms17101733 PMC5085763 27775595

[93] Lee SY , Ko KS . Effects of S‐adenosylmethionine and its combinations with taurine and/or betaine on glutathione homeostasis in ethanol‐induced acute hepatotoxicity. J Cancer Prev. 2016;21(3):164‐172. 27722142 10.15430/JCP.2016.21.3.164 PMC5051590

[94] Cavallaro RA , Fuso A , Nicolia V , Scarpa S . S‐adenosylmethionine prevents oxidative stress and modulates glutathione metabolism in TgCRND8 mice fed a B‐vitamin deficient diet. J Alzheimers Dis. 2010;20(4):997‐1002. 20413874 10.3233/JAD-2010-091666

[95] Bhatia P , Singh N . Homocysteine excess: delineating the possible mechanism of neurotoxicity and depression. Fundam Clin Pharmacol. 2015;29(6):522‐528. 26376956 10.1111/fcp.12145

[96] Bottiglieri T , Laundy M , Crellin R , Toone B , Carney M , Reynolds E . Homocysteine, folate, methylation, and monoamine metabolism in depression. J Neurol Neurosurg Psychiatry. 2000;69:228‐232. 10896698 10.1136/jnnp.69.2.228 PMC1737050

[97] Gao X , Hwang TC . Spatial positioning of CFTR's pore‐lining residues affirms an asymmetrical contribution of transmembrane segments to the anion permeation pathway. J Gen Physiol. 2016;147(5):407‐422. 27114613 10.1085/jgp.201511557 PMC4845689

[98] Moran O . The gating of the CFTR channel. Cell Mol Life Sci. 2017;74(1):85‐92. 27696113 10.1007/s00018-016-2390-z PMC11107742

[99] Zhu Q , Li H , Liu Y , Jiang L . Knockdown of CFTR enhances sensitivity of prostate cancer cells to cisplatin via inhibition of autophagy. Neoplasma. 2017;64(5):709‐717. 28592122 10.4149/neo_2017_508

[100] Zhang Y , Zhang Y , Tang J , et al. Oxymatrine inhibits homocysteine‐mediated autophagy via MIF/mTOR signaling in human umbilical vein endothelial cells. Cell Physiol Biochem. 2018;45(5):1893‐1903. 29510402 10.1159/000487912

[101] Chen Y , Zhang H , Liu H , Li K , Su X . Homocysteine up‐regulates ET receptors via suppression of autophagy in vascular smooth muscle cells. Microvasc Res. 2018;119:13‐21. 29601873 10.1016/j.mvr.2018.03.010

[102] Khayati K , Antikainen H , Bonder EM , et al. The amino acid metabolite homocysteine activates mTORC1 to inhibit autophagy and form abnormal proteins in human neurons and mice. FASEB J. 2017;31(2):598‐609. 28148781 10.1096/fj.201600915R

[103] Vendemiale G , Altomare E , Trizio T , et al. Effects of oral S‐adenosyl‐L‐methionine on hepatic glutathione in patients with liver disease. Scand J Gastroenterol. 1989;24(4):407‐415. 2781235 10.3109/00365528909093067

[104] Ko K , Yang H , Noureddin M , et al. Changes in S‐adenosylmethionine and GSH homeostasis during endotoxemia in mice. Lab Invest. 2008;88(10):1121‐1129. 18695670 10.1038/labinvest.2008.69 PMC4467989

[105] Villar VH , Merhi F , Djavaheri‐Mergny M , Duran RV . Glutaminolysis and autophagy in cancer. Autophagy. 2015;11(8):1198‐1208. 26054373 10.1080/15548627.2015.1053680 PMC4590661

[106] Mancilla H , Maldonado R , Cereceda K , et al. Glutathione depletion induces spermatogonial cell autophagy. J Cell Biochem. 2015;116(10):2283‐2292. 25833220 10.1002/jcb.25178

[107] Crespo JL , Hall MN . Elucidating TOR signaling and rapamycin action: lessons from *Saccharomyces cerevisiae* . Mol Biol Rev. 2002;66(4):579‐591, table of contents. 10.1128/MMBR.66.4.579-591.2002 PMC134654 12456783

[108] Stracka D , Jozefczuk S , Rudroff F , Sauer U , Hall MN . Nitrogen source activates TOR (target of rapamycin) complex 1 via glutamine and independently of Gtr/Rag proteins. J Biol Chem. 2014;289(36):25010‐25020. 25063813 10.1074/jbc.M114.574335 PMC4155668

[109] Duran RV , Hall MN . Glutaminolysis feeds mTORC1. Cell Cycle. 2012;11(22):4107‐4108. 23095634 10.4161/cc.22632 PMC3524199

[110] Duran RV , MacKenzie ED , Boulahbel H , et al. HIF‐independent role of prolyl hydroxylases in the cellular response to amino acids. Oncogene. 2013;32(38):4549‐4556. 23085753 10.1038/onc.2012.465 PMC3787797

[111] Handa AK , Fatima T , Mattoo AK . Polyamines: bio‐molecules with diverse functions in plant and human health and disease. Front Chem. 2018;6:10. 29468148 10.3389/fchem.2018.00010 PMC5807879

[112] Frostesjo L , Holm I , Grahn B , Page AW , Bestor TH , Heby O . Interference with DNA methyltransferase activity and genome methylation during F9 teratocarcinoma stem cell differentiation induced by polyamine depletion. J Biol Chem. 1997;272(7):4359‐4366. 9020157 10.1074/jbc.272.7.4359

[113] Hibasami H , Hoffman JL , Pegg AE . Decarboxylated S‐adenosylmethionine in mammalian cells. J Biol Chem. 1980;255(14):6675‐6678. 7391043

[114] Pegg AE , Wechter RS , Clark RS , Wiest L , Erwin BG . Acetylation of decarboxylated S‐adenosylmethionine by mammalian‐cells. Biochemistry. 1986;25(2):379‐384. 3006760 10.1021/bi00350a016

[115] Wagner J , Hirth Y , Piriou F , Zakett D , Claverie N , Danzin C . N‐acetyl decarboxylated S‐adenosylmethionine, a new metabolite of decarboxylated S‐adenosylmethionine – isolation and characterization. Biochem Bioph Res Commun. 1985;133(2):546‐553. 10.1016/0006-291x(85)90941-6 4084287

[116] Mato SCLJM . S‐Adenosylmethionine in cell growth, apoptosis and liver cancer. J Gastroenterol Hepatol. 2008;23:S73‐S77. 18336669 10.1111/j.1440-1746.2007.05289.x PMC2408691

[117] Sharma A , Gerbarg P , Bottiglieri T , et al. S‐adenosylmethionine (SAMe) for neuropsychiatric disorders: a clinician‐oriented review of research. J Clin Psychiatry. 2017;78(6):e656‐e667. 28682528 10.4088/JCP.16r11113 PMC5501081

[118] Zhang H , Liu Z , Ma S , et al. Ratio of S‐adenosylmethionine to S‐adenosylhomocysteine as a sensitive indicator of atherosclerosis. Mol Med Rep. 2016;14(1):289‐300. 27175774 10.3892/mmr.2016.5230

[119] Murin R , Vidomanova E , Kowtharapu BS , Hatok J , Dobrota D . Role of S‐adenosylmethionine cycle in carcinogenesis. Gen Physiol Biophys. 2017;36(5):513‐520. 29372684 10.4149/gpb_2017031

[120] Anstee QM , Day CP . S‐adenosylmethionine (SAMe) therapy in liver disease: a review of current evidence and clinical utility. J Hepatol. 2012;57(5):1097‐1109. 22659519 10.1016/j.jhep.2012.04.041

[121] Wilson A . S‐adenosyl methionine (SAMe) for depression in adults. Issues Ment Health Nurs. 2019;40(8):725‐726. 30633610 10.1080/01612840.2017.1392161

[122] Shea TB , Chan A . S‐adenosyl methionine: a natural therapeutic agent effective against multiple hallmarks and risk factors associated with Alzheimer’s disease. J Alzheimers Dis. 2008;13(1):67‐70. 18334758 10.3233/jad-2008-13107

[123] Skelly M , Hoffman J , Fabbri M , Holzman RS , Clarkson AB , Merali S . S‐adenosylmethionine concentrations in diagnosis of *Pneumocystis carinii* pneumonia. Lancet. 2003;361(9365):1267‐1268. 12699956 10.1016/S0140-6736(03)12984-4

[124] Sarris J , Byrne GJ , Bousman C , et al. Adjunctive S‐adenosylmethionine (SAMe) in treating non‐remittent major depressive disorder: an 8‐week double‐blind, randomized, controlled trial. Eur Neuropsychopharmacol. 2018;28(10):1126‐1136. 30115553 10.1016/j.euroneuro.2018.07.098

[125] Gregoire S , Millecamps M , Naso L , et al. Therapeutic benefits of the methyl donor S‐adenosylmethionine on nerve injury‐induced mechanical hypersensitivity and cognitive impairment in mice. Pain. 2017;158(5):802‐810. 28030474 10.1097/j.pain.0000000000000811

[126] Rutjes AW , Nüesch E , Reichenbach S , Jüni P . S‐Adenosylmethionine for osteoarthritis of the knee or hip. Cochrane Database Syst Rev 2009;(4):CD007321. 19821403 10.1002/14651858.CD007321.pub2 PMC7061276

[127] Galizia I , Oldani L , Macritchie K , et al. S‐adenosyl methionine (SAMe) for depression in adults. Cochrane Database Syst Rev. 2016;10(CD011286). 10.1002/14651858.CD011286.pub2 PMC6457972 27727432

[128] Cave M , Deaciuc I , Mendez C , et al. Nonalcoholic fatty liver disease: predisposing factors and the role of nutrition. J Nutr Biochem. 2007;18(3):184‐195. 17296492 10.1016/j.jnutbio.2006.12.006

[129] Yang HP , Ko K , Xia M , et al. Induction of avian musculoaponeurotic fibrosarcoma proteins by toxic bile acid inhibits expression of glutathione synthetic enzymes and contributes to cholestatic liver injury in mice. Hepatology. 2010;51(4):1291‐1301. 20146260 10.1002/hep.23471 PMC2908963

[130] Au AY , Hasenwinkel JM , Frondoza CG . Hepatoprotective effects of S‐adenosylmethionine and silybin on canine hepatocytes in vitro. Anim Physiol Anim Nutr. 2013;97(2):331‐341. 10.1111/j.1439-0396.2012.01275.x 22320165

[131] Chawla RK , Watson WH , Eastin CE , Lee EY , Schmidt J , McClain CJ . S‐adenosylmethionine deficiency and TNF‐alpha in lipopolysaccharide‐induced hepatic injury. Am J Physiol. 1998;275(1):G125‐129. 9655692 10.1152/ajpgi.1998.275.1.G125

[132] Yoon SY , Hong GH , Kwon HS , et al. S‐adenosylmethionine reduces airway inflammation and fibrosis in a murine model of chronic severe asthma via suppression of oxidative stress. Exp Mol Med. 2016;48(6):e236. 27256110 10.1038/emm.2016.35 PMC4929690

[133] Li TWH , Yang HP , Peng H , Xia M , Mato JM , Lu SC . Effects of S‐adenosylmethionine and methylthioadenosine on inflammation‐induced colon cancer in mice. Carcinogenesis. 2012;33(2):427‐435. 22159228 10.1093/carcin/bgr295 PMC3279046

[134] Feld JJ , Modi AA , El‐Diwany R , et al. S‐adenosyl methionine improves early viral responses and interferon‐stimulated gene induction in hepatitis C nonresponders. Gastroenterology. 2011;140(3):830–839.e3. 20854821 10.1053/j.gastro.2010.09.010 PMC3021477

[135] Chen H , Liu S , Ji L , et al. Folic acid supplementation mitigates Alzheimer’s disease by reducing inflammation: a randomized controlled trial. Mediators Inflamm. 2016;2016:5912146. 27340344 10.1155/2016/5912146 PMC4909909

[136] Morgan TR , Osann K , Bottiglieri T , et al. A phase II randomized, controlled trial of S‐adenosylmethionine in reducing serum α‐fetoprotein in patients with hepatitis C cirrhosis and elevated AFP. Cancer Prev Res (Phila). 2015;8(9):864‐872. 26130251 10.1158/1940-6207.CAPR-15-0029 PMC4560676

[137] Kim J , Lee EY , Koh E‐M , et al. Comparative clinical trial of S‐adenosylmethionine versus nabumetone for the treatment of knee osteoarthritis: an 8‐week, multicenter, randomized, double‐blind, double‐dummy, Phase IV study in Korean patients. Clin Ther. 2009;31(12):2860‐2872. 20110025 10.1016/j.clinthera.2009.12.016

[138] Chang CH , Qiu J , O'Sullivan D , et al. Metabolic competition in the tumor microenvironment is a driver of cancer progression. Cell. 2015;162(6):1229‐1241. 26321679 10.1016/j.cell.2015.08.016 PMC4864363

[139] Pua HH , Guo J , Komatsu M , He YW . Autophagy is essential for mitochondrial clearance in mature T lymphocytes. J Immunol. 2009;182(7):4046‐4055. 19299702 10.4049/jimmunol.0801143

[140] Stephenson LM , Miller BC , Ng A , et al. Identification of Atg5‐dependent transcriptional changes and increases in mitochondrial mass in Atg5‐deficient T lymphocytes. Autophagy. 2009;5(5):625‐635. 19276668 10.4161/auto.5.5.8133 PMC3737142

[141] Puleston DJ , Zhang HL , Powell TJ , et al. Autophagy is a critical regulator of memory CD8(+) T cell formation. eLife. 2014;3. 10.7554/eLife.03706 PMC4225493 25385531

[142] Schlie K , Westerback A , DeVorkin L , et al. Survival of effector CD8(+) T cells during influenza infection is dependent on autophagy. J Immunol. 2015;194(9):4277‐4286. 25833396 10.4049/jimmunol.1402571

[143] Xu XJ , Araki K , Li SZ , et al. Autophagy is essential for effector CD8(+) T cell survival and memory formation. Nat Immunol. 2014;15(12):1152‐1161. 25362489 10.1038/ni.3025 PMC4232981

[144] DeVorkin L , Pavey N , Carleton G , et al. Autophagy regulation of metabolism is required for CD8+ T cell anti‐tumor immunity. Cell Rep. 2019;27(2):502‐513.e505. 30970253 10.1016/j.celrep.2019.03.037

[145] Sahin E , Sahin M . Epigenetical Targeting of the FOXP3 gene by S‐adenosylmethionine diminishes the suppressive capacity of regulatory T cells ex vivo and alters the expression profiles. J Immunother. 2019;42(1):11‐22. 30407230 10.1097/CJI.0000000000000247

[146] Ma EH , Bantug G , Griss T , et al. Serine is an essential metabolite for effector T cell expansion. Cell Metab. 2017;25(2):345‐357. 28111214 10.1016/j.cmet.2016.12.011

[147] Wu Q , Chen X , Li J , Sun S . Serine and metabolism regulation: a novel mechanism in antitumor immunity and senescence. Aging Dis 2020 11(6). http://www.aginganddisease.org/EN/10.14336/AD.2020.0314 10.14336/AD.2020.0314 PMC7673844 33269112

[148] Cuyas E , Fernandez‐Arroyo S , Verdura S , et al. Metformin regulates global DNA methylation via mitochondrial one‐carbon metabolism. Oncogene. 2018;37(7):963‐970. 29059169 10.1038/onc.2017.367

[149] Zhong T , Men Y , Lu L , et al. Metformin alters DNA methylation genome‐wide via the H19/SAHH axis. Oncogene. 2017;36(17):2345‐2354. 27775072 10.1038/onc.2016.391 PMC5415944

[150] Cabreiro F , Au C , Leung KY , et al. Metformin retards aging in *C. elegans* by altering microbial folate and methionine metabolism. Cell. 2013;153(1):228‐239. 23540700 10.1016/j.cell.2013.02.035 PMC3898468

[151] Iida N , Dzutsev A , Stewart CA , et al. Commensal bacteria control cancer response to therapy by modulating the tumor microenvironment. Science. 2013;342(6161):967‐970. 24264989 10.1126/science.1240527 PMC6709532

[152] Zitvogel L , Ma Y , Raoult D , Kroemer G , Gajewski TF . The microbiome in cancer immunotherapy: diagnostic tools and therapeutic strategies. Science. 2018;359(6382):1366‐1370. 29567708 10.1126/science.aar6918

[153] Nijhout HF , Reed MC , Budu P , Ulrich CM . A mathematical model of the folate cycle: new insights into folate homeostasis. J Biol Chem. 2004;279(53):55008‐55016. 15496403 10.1074/jbc.M410818200

[154] Bytzer P , Talley NJ , Jones MP , Horowitz M . Oral hypoglycaemic drugs and gastrointestinal symptoms in diabetes mellitus. Aliment Pharmacol Ther. 2001;15(1):137‐142. 11136287 10.1046/j.1365-2036.2001.00896.x

[155] Diaz‐Morales N , Iannantuoni F , Escribano‐Lopez I , et al. Does metformin modulate endoplasmic reticulum stress and autophagy in type 2 diabetic peripheral blood mononuclear cells? Antioxid Redox Signal. 2018;28(17):1562‐1569. 29061071 10.1089/ars.2017.7409

[156] Wang Y , Xu W , Yan Z , et al. Metformin induces autophagy and G0/G1 phase cell cycle arrest in myeloma by targeting the AMPK/mTORC1 and mTORC2 pathways. J Exp Clin Cancer Res. 2018;37(1):63. 29554968 10.1186/s13046-018-0731-5 PMC5859411

[157] Bodmer M , Meier C , Krahenbuhl S , Jick SS , Meier CR . Long‐term metformin use is associated with decreased risk of breast cancer. Diabetes Care. 2010;33(6):1304‐1308. 20299480 10.2337/dc09-1791 PMC2875444

[158] Tsai HH , Lai HY , Chen YC , et al. Metformin promotes apoptosis in hepatocellular carcinoma through the CEBPD‐induced autophagy pathway. Oncotarget. 2017;8(8):13832‐13845. 28099155 10.18632/oncotarget.14640 PMC5355142

[159] Shi WY , Xiao D , Wang L , et al. Therapeutic metformin/AMPK activation blocked lymphoma cell growth via inhibition of mTOR pathway and induction of autophagy. Cell Death Dis. 2012;3:e275. 22378068 10.1038/cddis.2012.13 PMC3317343

[160] Heby O , Persson L , Rentala M . Targeting the polyamine biosynthetic enzymes: a promising approach to therapy of African sleeping sickness, Chagas’ disease, and leishmaniasis. Amino Acids. 2007;33(2):359‐366. 17610127 10.1007/s00726-007-0537-9

[161] Laukaitis CM , Gerner EW . DFMO: targeted risk reduction therapy for colorectal neoplasia. Best Pract Res Clin Gastroenterol. 2011;25(4–5):495‐506. 22122766 10.1016/j.bpg.2011.09.007 PMC3227870

[162] Hogarty MD , Norris MD , Davis K , et al. ODC1 is a critical determinant of MYCN oncogenesis and a therapeutic target in neuroblastoma. Can Res. 2008;68(23):9735‐9745. 10.1158/0008-5472.CAN-07-6866 PMC2596661 19047152

[163] Nilsson JA , Keller UB , Baudino TA , et al. Targeting ornithine decarboxylase in Myc‐induced lymphomagenesis prevents tumor formation. Cancer Cell. 2005;7(5):433‐444. 15894264 10.1016/j.ccr.2005.03.036

[164] Bachmann AS , Geerts D . Polyamine synthesis as a target of oncogenes. J Biol Chem. 2018;293(48):18757‐18769. 30404920 10.1074/jbc.TM118.003336 PMC6290138

[165] Basuroy UK , Gerner EW . Emerging concepts in targeting the polyamine metabolic pathway in epithelial cancer chemoprevention and chemotherapy. J Biochem. 2006;139(1):27‐33. 16428316 10.1093/jb/mvj022

[166] Mohammed A , Janakiram NB , Madka V , et al. Eflornithine (DFMO) prevents progression of pancreatic cancer by modulating ornithine decarboxylase signaling. Cancer Prev Res (Phila). 2014;7(12):1198‐1209. 25248858 10.1158/1940-6207.CAPR-14-0176 PMC4310684

[167] Raj KP , Zell JA , Rock CL , et al. Role of dietary polyamines in a phase III clinical trial of difluoromethylornithine (DFMO) and sulindac for prevention of sporadic colorectal adenomas. Br J Cancer. 2013;108(3):512‐518. 23340449 10.1038/bjc.2013.15 PMC3593561

[168] Sholler GLS , Ferguson W , Bergendahl G , et al. Maintenance DFMO increases survival in high risk neuroblastoma. Sci Rep. 2018;8(1):14445. 30262852 10.1038/s41598-018-32659-w PMC6160434

[169] Kreul SM , Havighurst T , Kim K , et al. A phase III skin cancer chemoprevention study of DFMO: long‐term follow‐up of skin cancer events and toxicity. Cancer Prev Res (Phila). 2012;5(12):1368‐1374. 23060038 10.1158/1940-6207.CAPR-12-0233 PMC3518692

[170] Bassiri H , Benavides A , Haber M , Gilmour SK , Norris MD , Hogarty MD . Translational development of difluoromethylornithine (DFMO) for the treatment of neuroblastoma. Transl Pediatr. 2015;4(3):226‐238. 26835380 10.3978/j.issn.2224-4336.2015.04.06 PMC4729051

[171] Travers M , Brown SM , Dunworth M , et al. DFMO and 5‐azacytidine increase M1 macrophages in the tumor microenvironment of murine ovarian cancer. Can Res. 2019;79(13):3445‐3454. 10.1158/0008-5472.CAN-18-4018 PMC6606334 31088836

